# RETRACTION: Evaluation of Wound Healing and Anti‐Inflammatory Activities of 80% Methanol Crude Extract and Solvent Fractions of *Trichilia dregeana* Sond (Meliaceae) Leaves in Mice

**DOI:** 10.1155/ecam/9874280

**Published:** 2025-12-18

RETRACTION: Daniel Gizachew Shewaye, Wubayehu Kahaliw, Tafere Mulaw Belete, Nejat Ahmed, “Evaluation of Wound Healing and Anti‐Inflammatory Activities of 80% Methanol Crude Extract and Solvent Fractions of *Trichilia dregeana* Sond (Meliaceae) Leaves in Mice,” *Evidence-Based Complementary and Alternative Medicine*, (2023): 9980866, https://doi.org/10.1155/2023/9980866.

The above article, published online on 19 January 2023 in Wiley Online Library (https://wileyonlinelibrary.com), has been retracted by John Wiley & Sons Ltd. The retraction has been agreed due to inconsistencies identified in the Figures, raised on PubPeer [1]. On further investigation, the below concerns have been identified:

• In Figure 5, there are repeated elements between the Day 16/simple ointment panel and the Day 10/10% w/w extract ointment panel in Figure 5 after resizing
• In Figure 10, panel i is a magnified and stretched part of panel c, despite intending to show different types of wound
• In Figure 7, the Day 4/10% w/w AQF ointment panel appears similar to the following:
  ◦ The Day 12/5% w/w extract ointment panel in Figure 9
  ◦ The Day 4/5% w/w NHF ointment panel Figure 7
• In Figure 7, the Day 10/10% w/w AQF ointment panel appears similar to the Day 20/simple ointment panel in Figure 9
• In Figure 7, the Day 16/5% w/w NHF ointment panel appears similar to the Day 16/10% w/w NHF ointment panel in Figure 7
• In Figure 7, the Day 4/10% w/w NHF ointment panel appears similar when rotated to the Day 2/5% w/w CEO panel in Figure 8 of [2].
• In Figure 7, the Day 16/10% w/w NHF ointment panel appears similar to the Day 10/10% w/w AQF panel in Figure 10 of [2].
• In Figure 9, the Day 20 simple ointment panel appears identical to the Day 20/nitrofurazone 0.25 w/v ointment panel in Figure 9
• In Figure 9, the Day 12/nitrofurazone 0.25 w/v ointment panel appears identical to the Day 10/10% w/w HF panel in Figure 10 of [2].

The authors did not provide a response to the above concerns. The results and conclusions are therefore deemed unreliable, and the article is retracted.

The authors did not respond to indicate agreement or disagreement with the retraction.
